# Cholesterol depletion decreases adhesion of non-small cell lung cancer cells to E-selectin

**DOI:** 10.1152/ajpcell.00197.2020

**Published:** 2023-07-03

**Authors:** Amina Mohammadalipour, Christian A. Showalter, Harrison T. Muturi, Amir M. Farnoud, Sonia M. Najjar, Monica M. Burdick

**Affiliations:** ^1^Department of Physics and Department of Biomedical Engineering, School of Science and Engineering, Saint Louis University, Saint Louis, Missouri, United States; ^2^Department of Biological Sciences, College of Arts and Sciences, Ohio University, Athens, Ohio, United States; ^3^Department of Biomedical Sciences, Heritage College of Osteopathic Medicine, Ohio University, Athens, Ohio, United States; ^4^Department of Chemical and Biomolecular Engineering, Russ College of Engineering and Technology, Ohio University, Athens, Ohio, United States; ^5^Diabetes Institute, Heritage College of Osteopathic Medicine, Ohio University, Athens, Ohio, United States

**Keywords:** cell adhesion, cholesterol, lipid microdomain, lung cancer, membrane fluidity

## Abstract

Lipid microdomains, ordered membrane phases containing cholesterol and glycosphingolipids, play an essential role in cancer cell adhesion and ultimately metastasis. Notably, elevated levels of cholesterol-rich lipid microdomains are found in cancer cells relative to their normal counterparts. Therefore, alterations of lipid microdomains through cholesterol modulation could be used as a strategy to prevent cancer metastasis. In this study, methyl-beta-cyclodextrin (MβCD), sphingomyelinase (SMase), and simvastatin (Simva) were used to investigate the effects of cholesterol on the adhesive behaviors of four non-small cell lung cancer (NSCLC) cell lines (H1299, H23, H460, and A549) and a small cell lung cancer (SCLC) cell line (SHP-77) on E-selectin, a vascular endothelial molecule that initiates circulating tumor cell recruitment at metastatic sites. Under hemodynamic flow conditions, the number of adherent NSCLC cells on E-selectin significantly decreased by MβCD and Simva treatments, whereas SMase treatment did not show a significant effect. Significant increases in rolling velocities were detected only for H1299 and H23 cells after MβCD treatment. In contrast, cholesterol depletion did not affect SCLC cell attachment and rolling velocities. Moreover, cholesterol depletion by MβCD and Simva induced CD44 shedding and resulted in an enhanced membrane fluidity in the NSCLC cells, whereas it did not affect the membrane fluidity of the SCLC cells which lacked detectable expression of CD44. Our finding suggests that cholesterol regulates the E-selectin-mediated adhesion of NSCLC cells by redistributing the CD44 glycoprotein and thus modulating the membrane fluidity.

**NEW & NOTEWORTHY** This study investigates the effects of cholesterol on the adhesive behaviors of lung cancer cells in recruitment at metastatic sites. Using cholesterol-modulating compounds, we found that reducing cholesterol decreases the adhesion of non-small cell lung cancer (NSCLC) cells while having no significant effect on small cell lung cancer (SCLC) cells. The study suggests that cholesterol regulates NSCLC cell metastasis by redistributing the adhesion proteins on the cells and modulating cells’ membrane fluidity.

## Graphical abstract

**Figure:**
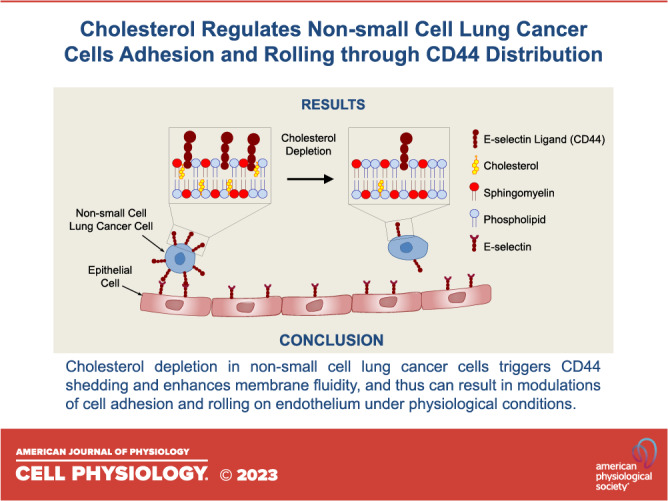

## INTRODUCTION

Despite numerous advances in cancer research, lung cancer has remained one of the most common malignancies and a primary cause of cancer-related mortality worldwide (1, 2). Notoriously heterogeneous, lung cancer is mainly divided into two histopathological groups as non-small cell lung cancer (NSCLC, ∼80%–85%) and small cell lung cancer (SCLC, ∼15%–20%) (3). The 2-year and 5-year survival rates are ∼48% and 28% for NSCLC and 17% and 7% for SCLC, respectively (4). The low survival rates are mainly related to the distinct-stage diagnosis relative to the metastatic site (1, 2). Metastasis of tumor cells, as a mimic of leukocytes’ migration to a center of inflammation, has been implicated to be initiated by cell tethering and rolling on the vascular endothelium (5–7). This process is mediated by selectin-ligand interactions between the endothelial and cancer cells. Transmembrane glycoproteins expressed on cancer cells promote bond formations with adhesive molecules on the endothelium such as E-selectin, which is optimally effective under the influence of shear stress (8). Recently, our laboratory used a novel technique, dynamic biochemical tissue analysis, to detect functional E-selectin ligands in human lung cancer tissues, thereby implying that selectin-ligand interactions may mediate metastasis to distant sites (9). Most reported selectin ligands are sialofucosylated glycoproteins, including specific glycoforms of CD44 (10). On its own, CD44 is a highly diversified transmembrane glycoprotein consisting of several isoforms extensively expressed on cancerous cells such as lung (11, 12), renal (13), gastric (14), colon (15), pancreatic (16), and breast cancers (10), leading to numerous roles in cell adhesion, proliferation, and signaling. CD44 has been shown to be localized in the cholesterol- and sphingomyelin-rich compartments on the cell plasma membrane, commonly referred to as lipid microdomains (17). Cholesterol and sphingomyelin are positively correlated and colocalized in various membrane functions, such as lipid microdomain formation, membrane stability, and modulating phosphorylation cascades initiating from membrane-bound proteins (18–20). In addition, recent studies have shown an indispensable role of membrane cholesterol in cancer cell migration and invasion through the regulation of adhesive glycoproteins on the cell membrane (21–23).

That said, it is not clear how cholesterol or sphingomyelin may affect lung cancer cells rolling on vascular endothelial E-selectin. Cholesterol, a sterol that makes up about one-third of the lipid content of the plasma membrane, plays an essential role in membrane properties such as fluidity and permeability as well as maintaining the architecture and stability of the cell membrane (24, 25). In agreement with the early observations on the cholesterol content of malignant tumor cells (26), recent studies have confirmed an accumulation of cholesterol on the surface of cancer cells (27, 28). The abnormal accumulation of cholesterol is also associated with tumor growth and survival (20, 29). Consequently, manipulating lipid microdomains through cholesterol depletion and inhibition reagents, such as methyl-β-cyclodextrin (MβCD) and simvastatin (Simva), have been extensively used to investigate the physiologic function of cholesterol in maintaining the integrity of the cell plasma membrane (30–32). Although MβCD is able to selectively extract cholesterol from the membrane of the cell without binding or inserting into the plasma membrane (33), Simva lowers cholesterol by inhibiting the key enzyme of cholesterol biosynthesis, HMG-CoA reductase (34). Moreover, lipid microdomain disruption can be approached through the enzymatic breakdown of sphingomyelin by SMase, which has also been reported to regulate cholesterol distribution within the membrane (35).

Given recent reports demonstrating that nanoscale liposomes targeting E-selectin ligands on circulating cancer cells may lead to better patient outcomes (36, 37), there is a greater need to understand fundamental selectin/selectin-ligand mediated cancer cell adhesion during metastasis. The study herein examined the effects of cholesterol depletion, inhibition, and disruption by MβCD, Simva, and SMase treatments on cell adhesion and rolling of four different NSCLC cell lines (H1299, H23, H460, and A549) and an SCLC cell line (SHP-77) under simulated hemodynamic shear stress in an E-selectin-coated parallel-plate flow chamber.

## MATERIAL AND METHODS

### Cell Culture and Size Measurements

H23, H1299, H460, and SHP-77 lung cancer cell lines were obtained from the American Type Culture Collection (ATCC, Manassas, VA) and were cultured in RPMI-1640 medium (ATCC) supplemented with 10% fetal bovine serum (FBS). The A549 lung cancer cell line from ATCC was cultured in F-12K medium (ATCC) supplemented with 10% FBS. The cells were maintained at 37°C and 5% CO_2_ in an incubator and subcultured every 2–3 days. A Scepter handheld automated cell counter (Millipore, Burlington, MA) was used to measure the diameter and volume of the NSCLC and SCLC cell lines following the manufacturer’s protocol.

### Cell Proliferation Assay (MTS)

H1299 lung cancer cells were seeded at 3 × l0^4^ cells/well in a 96-well plate (Corning) with a final volume of 200 μL/well. Multiple concentrations of MβCD, Simva, and SMase (all from Sigma Aldrich, St. Louis, MO) were used for cholesterol depletion. Dimethyl sulfoxide (DMSO, Millipore, Burlington, MA) was used as a control for Simva, and Dulbecco’s phosphate buffered saline with calcium and magnesium (DPBS^+^, HyClone, South Logan, UT) was used as a control for MβCD and SMase. After the treatments, 20 µL of MTS reagent (Promega, Madison, WI) was added into each well and incubated for 3 h at 37°C. The absorbance of nontreated and treated cells was measured at 490 nm using a Synergy2 plate reader (BioTek, Winooski, VT).

### Cholesterol Depletion, Inhibition, and Disruption Treatments

Cells for treatment were seeded at l0^6^ cells in a six-well plate. For MβCD treatment, the cells were incubated with 10 mM MβCD for 30 min (38). Simva was added to the cells at the concentration of 10 µM for 20 h (39). For the treatment with SMase, first, the cells were kept in serum-free media for 15 min in a CO_2_ incubator at 37°C, then SMase was added at a final concentration of 100 mU/mL for 20 min (35). All the cell treatments were performed in a CO_2_ incubator at 37°C.

### Cell Viability Assay

Lung cancer cells were seeded at the density of l0^6^ cells in six-well plates. The cells were kept nontreated or treated with MβCD (10 mM for 30 min), Simva (10 µM for 20 h), or SMase (100 mU/mL for 20 min) (35, 38, 39). DMSO was used as the control for Simva, and DPBS^+^ was used as the control for MβCD and SMase. Ten microliters of cell suspensions were stained with 10 µL of trypan blue, and the viability of the cells was measured by dye exclusion using a hemocytometer and light microscopy.

### Amplex Red Cholesterol Assay

The Amplex Red cholesterol assay kit (Molecular Probes, Eugene, OR) was used for cholesterol content quantification in cells before and after treatments, based on a previously published method for plasma membrane cholesterol in intact cells (40). Briefly, the cells were plated at the density of 10^6^ cells/well in a six-well plate overnight. Nontreated and treated cell samples were diluted in 1× reaction buffer and seeded in a 96-well plate (50 µL ≈ 10^5^ cells/well) in triplicate. Then, 50 μL of 300 μM Amplex Red reagent containing 2 U/mL horseradish peroxidase (HRP), 2 U/mL cholesterol oxidase, and 0.2 U/mL cholesterol esterase was added to each sample and were incubated for 30 min at 37°C in dark. Fluorescence was measured in the Synergy2 fluorescence plate reader using excitation in the range of 530–560 nm and emission detection at 590 nm.

### Antibodies

Mouse anti-human CD44 antibody (clone 2C5; Cat. No. BBA10), mouse IgG_2A_ isotype control (Cat. No. MAB0031), and anti-mouse IgG fluorescein-conjugated antibody (Cat. No. F0103B) were purchased from R&D Systems (Minneapolis, MN). Mouse anti-human sLe^A^ antibody (clone 116-NS-19-9; Cat. No. MA5-12421) and PE-conjugated mouse IgG_1_ isotype control (Cat. No. 12-4714-42) were purchased from Invitrogen (Waltham, MA). Mouse anti-human sLe^X^ antibody (clone CSLEX-1; Cat. No. 551344), PE-conjugated rat anti-human cutaneous lymphocyte (clone HECA-452; Cat. No. 563962), and PE-conjugated rat IgM isotype control (Cat. No. 553943) were obtained from BD Biosciences (San Jose, CA).

### Flow Cytometry

Using a FACSAria special order research product flow cytometer/sorter (BD Biosciences, San Jose, CA), surface molecular expression levels of CD44 (2C5), sLe^A^ (116-NS-19-9), sLe^X^ (CSLEX-1), and HECA-452 antigen were analyzed on the nontreated and treated lung cancer cell lines. Cell suspensions at a density of 10^7^ cells/mL were incubated with 10 μg/mL of antibodies or with their matched isotype control antibodies for 30–45 min at 4°C. Cells were washed three times with 0.1% bovine serum albumin (BSA) in DPBS^+^ and were incubated with secondary antibodies for 30 min at 4°C. Cells were washed again twice with 0.1% BSA/DPBS^+^, followed with one DPBS^+^ wash, and then were resuspended in DPBS^+^ before running through the flow cytometer.

### Flow Chamber Adhesion Assay

Cell adhesion assessment of the panel of lung cancer cell lines was performed on recombinant human E-selectin/Fc chimera (rhE-Selectin Fc/Chimera; R&D Systems, Minneapolis, MN). Immobilized rhE-selectin substrate was made at 10 μg/mL of the protein incubated on a Petri dish at 4°C overnight. The substrate was blocked by 1% BSA for 2 h at 4°C. Nontreated and treated cancer cells suspended at 10^6^ cells/mL in 0.1% BSA/DPBS^+^ were added to a syringe connected by plastic tubing to the parallel plate flow chamber (41). The aqueous cell solution was pulled through the gasket by an automated syringe pump set at a volumetric flow rate corresponding to 0.8 dyne/cm^2^ wall shear stress. The flow chamber was placed on an inverted microscope, which was equipped with a camera connected to a PC for image acquisition. The number of attached cancer cells to E-selectin Fc chimera coated surfaces as well as the rolling velocities were obtained by Image J software (National Institutes of Health, Bethesda, MD) (41).

### Quantitative Reverse Transcriptase Polymerase Chain Reaction

Total RNA was isolated from lung cancer cells using an RNeasy plus mini kit (Qiagen, Valencia, CA) following the manufacturer’s protocol. Genomic DNA was separated from total RNA using a gDNA spin column, and genomic DNA was subsequently disposed. Total RNA purity and concentration were evaluated using a NanoVue Plus (GE Healthcare Biosciences, Piscataway, NJ), and 0.7 µg of total RNA was reverse transcribed to cDNA using a high-capacity reverse transcription kit (Applied Biosystems, Foster City, CA). The quantitative reverse transcriptase polymerase chain reaction (qRT-PCR) analysis was performed using a Step One Plus Real-Time PCR instrument (Applied Biosystems) using primers for CD44s (forward primer: 5′- CCTCCAGTGAAAGGAGCAGCAC-3′; reverse primer: 5′- GTGTCTTGGTCTCTGGTAGCAGGGAT-3′) and RPL13A (forward primer: 5′- GAGGCCCCTACCACTTCC-3′; reverse primer: 5′- AACACCTTGAGACGGTCCAG-3′) (Integrated DNA Technologies, Coralville, IA) and SYBR green PCR master mix (Applied Biosystems). The mRNA expression data were normalized to housekeeping gene ribosomal protein L13A (RPL13A) (42, 43), and relative mRNA expression was quantified using the Pfaffl method (44). All cDNA samples were loaded in triplicate.

### Western Blotting

Proteins were extracted from cultured cells using lysis buffer containing 150 mM NaCl-50 mM HEPES (pH 7.6), 0.02% sodium azide, 1% Triton X-100, 50 mM NaF, PMSF, Na_3_VO_4_, and protease Inhibitor Cocktail Tablet (Roche Diagnostics, Penzberg, Germany). The protein lysates were denatured by boiling in sodium dodecyl sulfate (SDS) buffer for 3 min. An equal amount of protein (10–30 µg) was resolved by 7% SDS-polyacrylamide gels, then transferred onto a nitrocellulose membrane (Bio-Rad Laboratories, Hercules, CA). After blocking with 3%–5% dry milk or BSA dissolved in TBST (Tris-buffered saline, pH 7.2, 0.1% Tween 20), the membrane was incubated with primary antibody (1:1,000) against CD44 (2C5) at 4°C overnight. Next day, the membrane was washed three times with TBST and incubated with the secondary antibody for 1 h at room temperature. For normalization, the membrane was reprobed with monoclonal antibodies against tubulin (Cell Signaling Technology, Danvers, MA). The protein was visualized using enhanced chemiluminescence (ECL) detection (Amersham Pharmacia, Sunnyvale, CA).

### Membrane Fluidity

Membrane fluidity was assessed using pyrenedecanoic acid (PDA; Membrane Fluidity Kit; Marker Gene Technologies, Eugene, OR). Cells (∼3 × 10^6^) were used for each independent experiment in triplicate. The labeling solution contained 20 µM of fluorescent lipid reagent and a final concentration of 0.08% Pluronic F127 in media. The cells were incubated with the labeling solution at room temperature for 20 min in dark. The cells were then washed three times with media and plated in a 96-well plate at a final volume of 200 mL. Fluorescence intensities were measured at 420 nm (monomer) and 460 nm (excimer) emissions for excitation at 360 nm using the Synergy2 fluorescence plate reader.

### Statistics

All the experiments were repeated at least three times under independent conditions. Data are represented as means ± SE (standard error). Statistical significances (*P* < 0.05) between nontreated and treated cells were evaluated via one-way ANOVA with Tukey’s honest significant difference (HSD) post hoc test.

## RESULTS

### NSCLC and SCLC Average Volume and Cholesterol Content

The Amplex Red cholesterol assay kit was used to quantify the plasma membrane cholesterol content of the cells (40). The results showed that all the NSCLC cell lines had significantly higher levels of cholesterol compared with the SCLC cell line (Table 1). Comparisons between the NSCLC cell lines revealed that H1299 cells had the highest membrane cholesterol content, followed by H23 cells. H460 and A549 cell lines together were found to have significantly lower membrane cholesterol content compared with the other NSCLC cell lines while still containing higher cholesterol compared with the SCLC cell line. The size of the tumor cells might affect these measurements, as well as some of their adhesion characteristics and metastatic potential. Therefore, the average volumes of the lung cancer cell lines were measured in a Scepter handheld automated cell counter. The average diameter of the small lung cancer cells, SHP-77, was roughly twofold less than that of the non-small lung cancer cells (Table 1). Thus, considering the volume of the cells, the SHP-77 cell line had the highest content of cholesterol per cell volume, whereas H1299 had the smallest amount of cholesterol per cell volume.

**Table 1.** Average volume and cholesterol content of NSCLC and SCLC cell lines (±SE)

Cell Line	Cholesterol, µg/10^6^ cells	Diameter, µm	Volume, µm^3^	Cholesterol/Volume, fg/µm^3^	Cell Line Classification
H1299	33.24 ± 2.0	19.25 ± 0.39	29,948.50 ± 3,084.71	1.11 ± 0.03	non-small cell carcinoma
H23	29.81 ± 1.0	17.92 ± 0.31	24,160.91 ± 1,252.11	1.24 ± 0.02	non-small cell adenocarcinoma
H460	25.79 ± 3.0	15.84 ± 0.42	16,708.17 ± 2,285.72	1.55 ± 0.07	non-small cell carcinoma
A549	26.21 ± 3.0	15.58 ± 0.37	15,876.08 ± 1,166.77	1.65 ± 0.08	non-small cell carcinoma
SHP-77	9.30 ± 1.0	8.30 ± 0.67	2,489.96 ± 550.37	3.75 ± 0.17	small cell carcinoma

Values are means ± SE. NSCLC, non-small cell lung cancer; SCLC, small cell lung cancer.

### Cell Lines Were Viable under the Selected Concentrations of Treatments

**Figure 1.**
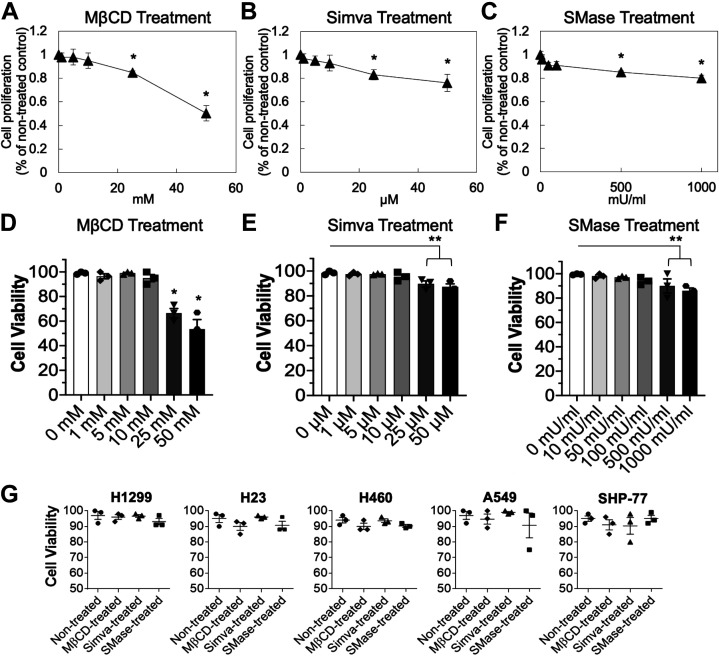
MTS cell proliferation and viability assays. H1299 cells were treated with various concentrations of MβCD for 30 min (*A*), Simva for 20 h (*B*), or SMase for 20 min (*C*). Cell proliferation was tested by MTS assay. Results are the means ± SE over *n* = 3 independent experiments. Statistical analysis was performed via one-way ANOVA with Tukey’s HSD post hoc test. *Significant difference from other concentrations (*P* < 0.05). *D–F*: using the trypan blue assay, the cell viability of H1299 cells was tested for different concentrations of MβCD for 30 min, Simva for 20 h, and SMase for 20 min. Results are the means ± SE over *n* = 3 independent experiments. Statistical analysis was performed via one-way ANOVA with Tukey’s HSD post hoc test. *Significant difference from the other concentrations, and **Significant difference from the rest of the concentrations but not between each other (*P* < 0.05). *G*: using the trypan blue assay, the viability of all lung cancer cell lines was tested upon treatment with 10 mM of MβCD for 30 min, 10 µM of Simva for 20 h, or 100 mU/mL of SMase for 20 min. Results are the means ± SE over *n* = 3 individual experiments. HSD, honest significant difference; MβCD, methyl-beta-cyclodextrin.

The MTS cell proliferation assay is a colorimetric method for the sensitive quantification of viable cells. The assay was performed for multiple concentrations of MβCD for 30 min, Simva for 20 h, and SMase for 20 min to examine the potential cytotoxicity of the treatments to H1299 lung cancer cell line (Fig. 1, *A–C*). Time points used in the MTS cell proliferation assay and subsequent experiments were selected based on previous studies that used MβCD (38), Simva (39), or SMase (35) for cholesterol depletion or disruption. In addition, the viability of treated and nontreated H1299 cells was measured by trypan blue exclusion assay using a hemocytometer (Fig. 1, *D–F*).

The results showed that the highest concentrations of the drugs in which the cells were more than 90% proliferative and viable were at 10 mM, 10 µM, and 100 mU/mL for MβCD, Simva, and SMase, respectively. Therefore, the viability of all the studied lung cancer cell lines was examined under the selected three treatment concentrations; 10 mM of MβCD for 30 min, 10 µM of Simva for 20 h, and 100 mU/mL of SMase for 20 min (Fig. 1*G*). DMSO was used as the control for Simva treatment, and DPBS^+^ was used as the control for MβCD and SMase treatments. As the results of the DMSO and DPBS^+^ controls were not significantly different from nontreated cells, for simplicity of the graphs, herein, we only present the nontreated cells as the control. Using the trypan blue assay, all the cell lines were ≥ 90% viable under these treatments; thus, the mentioned concentration and time point treatments were used throughout different assays in this study.

### Cholesterol Depletion of the Plasma Membrane

**Figure 2.**
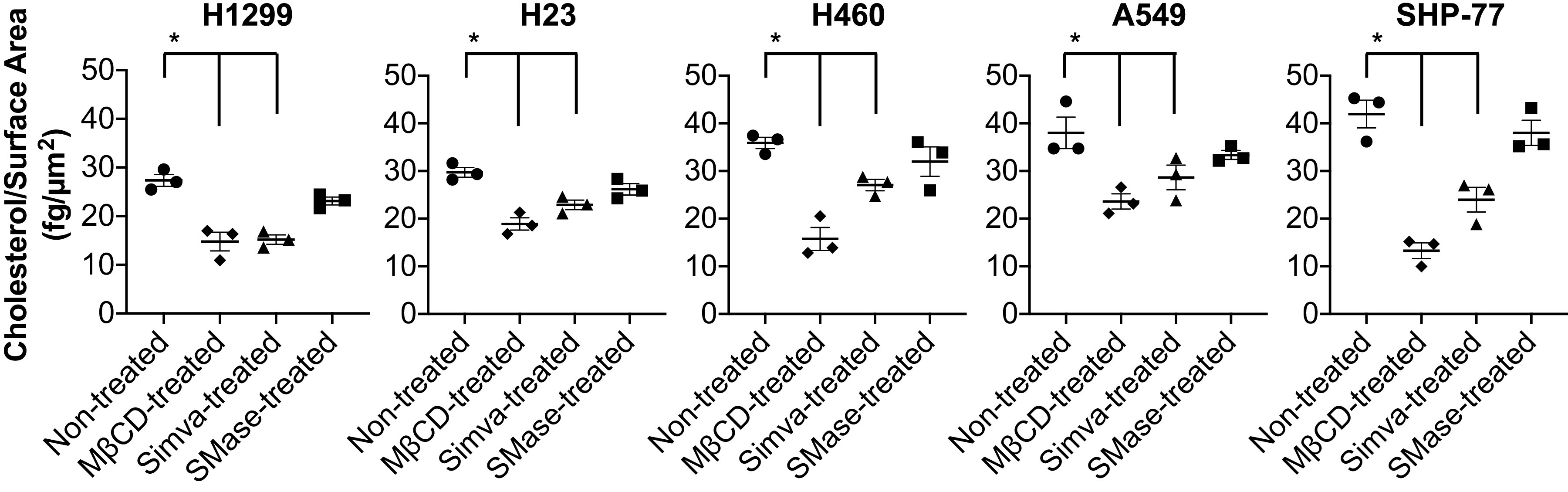
Membrane cholesterol content of lung cancer cell lines per surface area before and after cholesterol depletion treatments. Membrane cholesterol per surface area significantly reduced for all the lung cancer cell lines upon MβCD and Simva treatments. Values are means ± SE for *n* = 3 independent experiments. Statistical analysis was performed by one-way ANOVA with Tukey’s HSD post hoc comparison. *Statistical significance compared with nontreated cells (*P* < 0.05). HSD, honest significant difference; MβCD, methyl-beta-cyclodextrin.

To investigate the effects of cholesterol depletion on cell rolling and adhesion under shear flow, first the efficacy of cholesterol depletion using the selected concentrations of MβCD (10 mM for 30 min), Simva (10 µM for 20 h), and SMase (100 mU/mL for 20 min) was examined by measuring membrane cholesterol content of the lung cancer cells before and after treatments. Upon treatments with MβCD and Simva, the level of membrane cholesterol per surface area was decreased for all the studied lung cancer cell lines, whereas SMase did not affect the level of the cholesterol content per surface area in any of the cell lines (Fig. 2).

### H1299 and H23 NSCLC Cell Lines Express Classical Glycans

**Figure 3.**
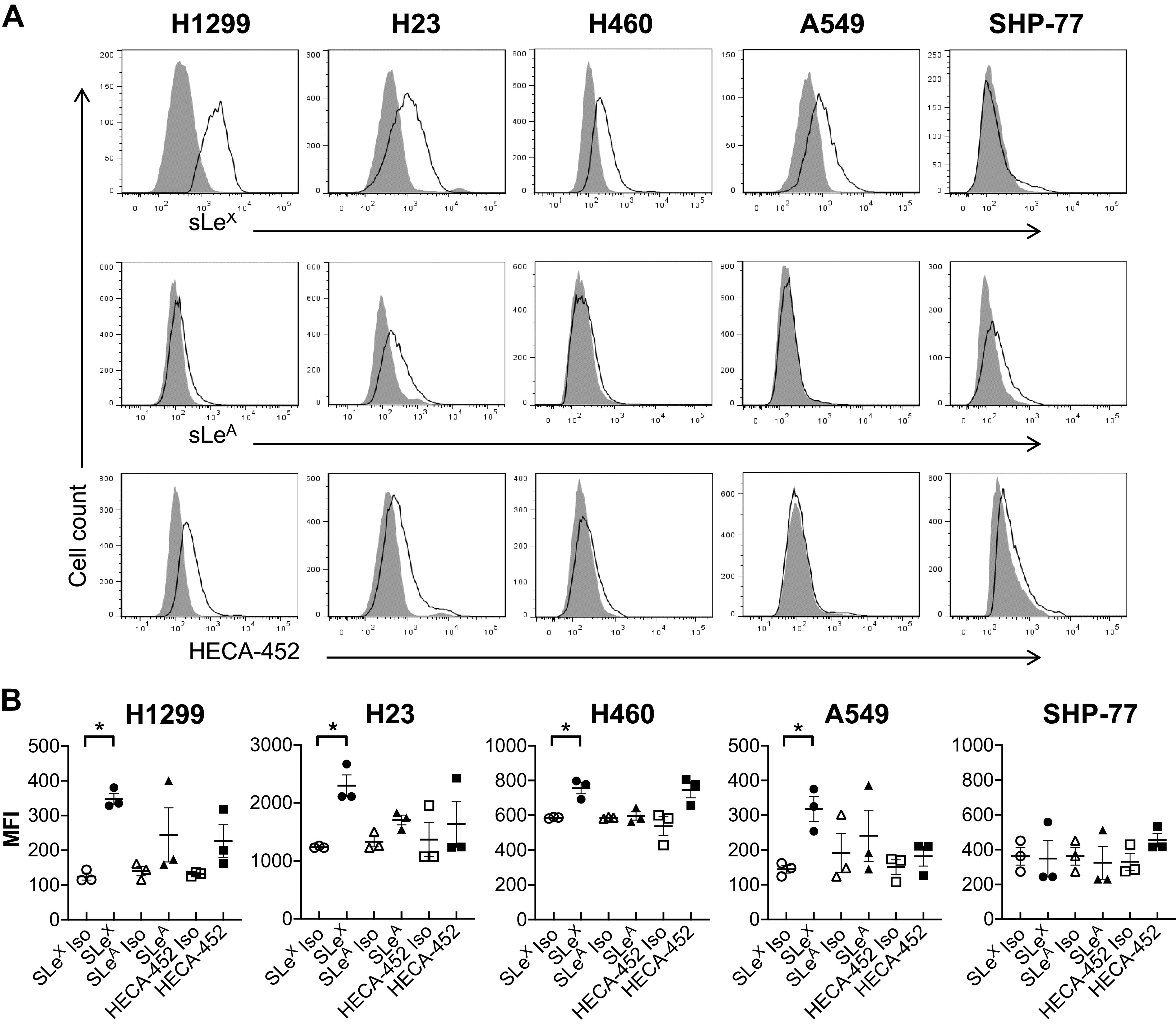
Lung cancer cells were probed for a variety of known selectin ligand epitopes via flow cytometry. *A*: histograms are representative of *n* = 3 independent experiments. Isotype control is represented by the filled region, whereas specific antibody expression is shown with unfilled lines. *B*: statistical analysis of the mean fluorescence intensities (MFIs) showed that all NSCLC cell lines were strongly positive for sialofucosylated sLe^X^. Only H23 weakly expressed sLe^A^, and only H1299 and H23 weakly expressed HECA-452 reactive epitopes. Values are means ± SE for *n* = 3 independent experiments. Statistical analysis was performed by one-way ANOVA with Tukey’s HSD post hoc test comparison. *Statistical significance compared with the relative isotype control (*P* < 0.05). HSD, honest significant difference; NSCLC, non-small cell lung cancer.

It is known that E-selectin initially recognizes sialylated and fucosylated epitopes of glycans on E-selectin ligands. Thus, lung cancer cell expression of glycans correlating with E-selectin ligand activity was characterized by flow cytometry. Labeling the cells with mAbs against sLe^X^, sLe^A^, and HECA-452 antigen revealed that all NSCLC cells strongly expressed sLe^X^ (Fig. 3). Only H23 was weakly positive for sLe^A^. None of the studied lung cancer cell lines expressed HECA-452, except for H1299 and H23 cells in which weak reactivity levels with HECA-452 were observed (Fig. 3). The results suggest that only H1299 and H23 cells express classical glycans involved in E-selectin mediated adhesion, whereas the rest of the cell lines might possess other glycans that bind to E-selectin.

### E-Selectin Dependent Attachment of NSCLC Cells Decreases with Cholesterol Depletion

**Figure 4.**
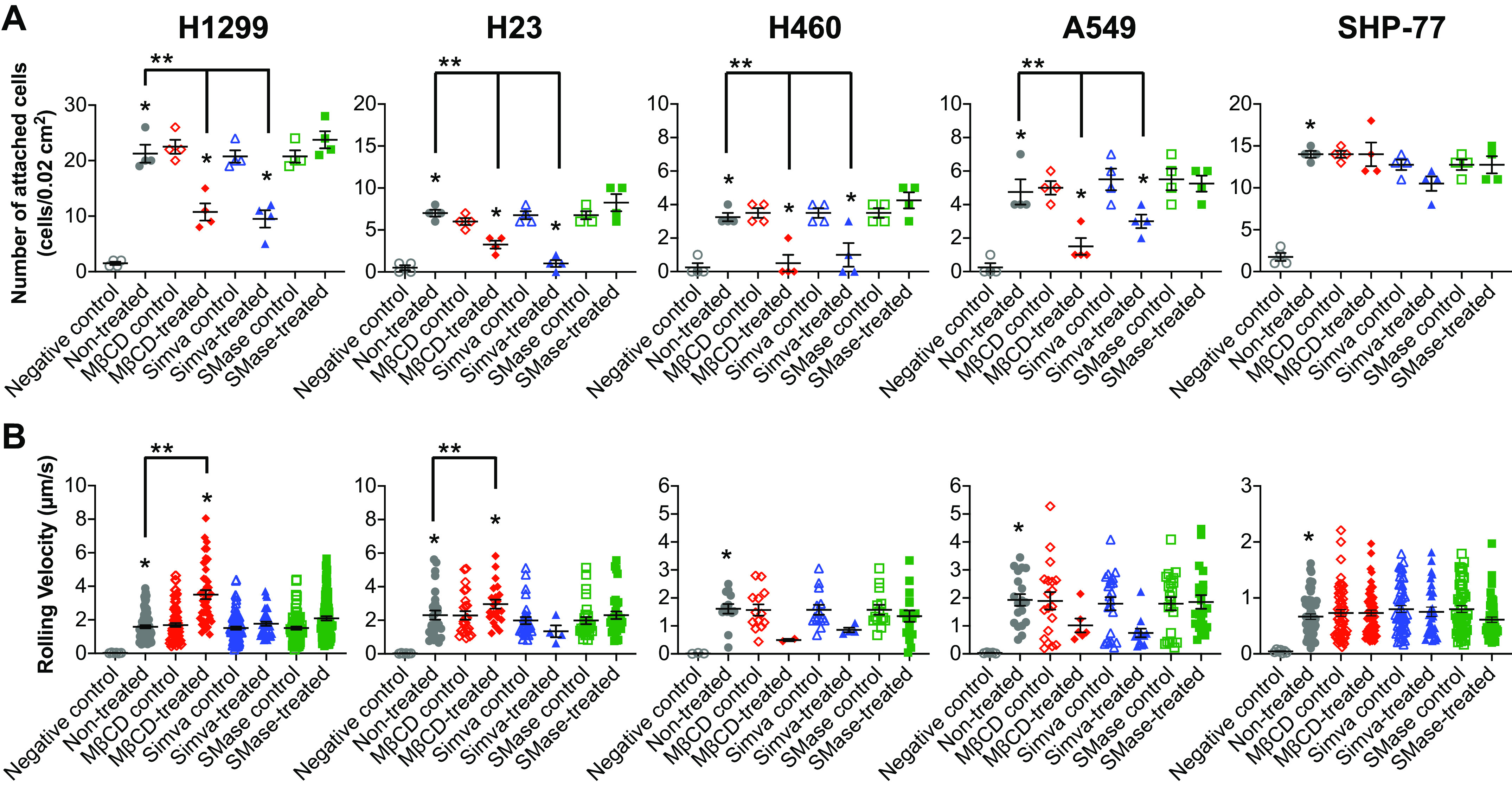
Effect of cholesterol depletion on cell adhesion and rolling velocity of lung cancer cell lines on E-selectin-coated surfaces under physiological conditions in a flow chamber. *A*: of attached cells decreased for all the NSCLC cells upon MβCD and Simva treatments, whereas the SCLC cell line remained unaffected. *B*: the rolling velocities of H1299 and H23 cell lines significantly increased only through MβCD treatment. Values are means ± SE for *n* = 4 independent experiments. Statistical analysis was performed by one-way ANOVA with Tukey’s HSD post hoc test. *Statistical significance compared with the relative control (*P* < 0.05). **Statistical difference compared with nontreated cells (*P* < 0.05). HSD, honest significant difference; MβCD, methyl-beta-cyclodextrin; NSCLC, non-small cell lung cancer; SCLC, small cell lung cancer.

The effect of cell membrane cholesterol depletion on E-selectin-mediated adhesion was tested under physiological flow conditions using a parallel plate flow chamber assay (41). Nontreated and treated lung cancer cells or their relative controls (DMSO for Simva-treated and DPBS^+^ for MβCD-treated and SMase-treated) were perfused over E-selectin-coated substrates. As a negative control, nontreated cells were also perfused on rhIgG-coated substrates (nontreated control in Fig. 4) for which no adhesion was observed, indicating all the cell attachments were E-selectin dependent. In addition, no significant differences were found between nontreated cells and the controls for treatments, thus it would be relevant if we considered nontreated cells as the control in the statistical analysis. The results showed that H1299 and SHP-77 cell lines were the first and second highest adherent cell lines, respectively (Fig. 4*A*). However, the SCLC cells were found to transit with significantly lower velocities compared with the NSCLC cells (Fig. 4*B*).

Cholesterol depletion and inhibition through MβCD and Simva resulted in a significant decrease in the number of attached cells to E-selectin substrates for all the NSCLC lines (Fig. 4*A*). In addition, MβCD but not Simva, significantly increased the rolling velocities of H1299 and H23 cell lines (Fig. 4*B*). In contrast, cholesterol depletion showed no effect on the number of attached cells and rolling velocities of the SCLC cells (Fig. 4, *A* and *B*). Overall, the data showed that cholesterol depletion from the plasma membrane could affect the successful adhesion of NSCLC cells, but not SCLC cells, to E-selectin.

### NSCLC Cells but Not SCLC Cells Express CD44

**Figure 5.**
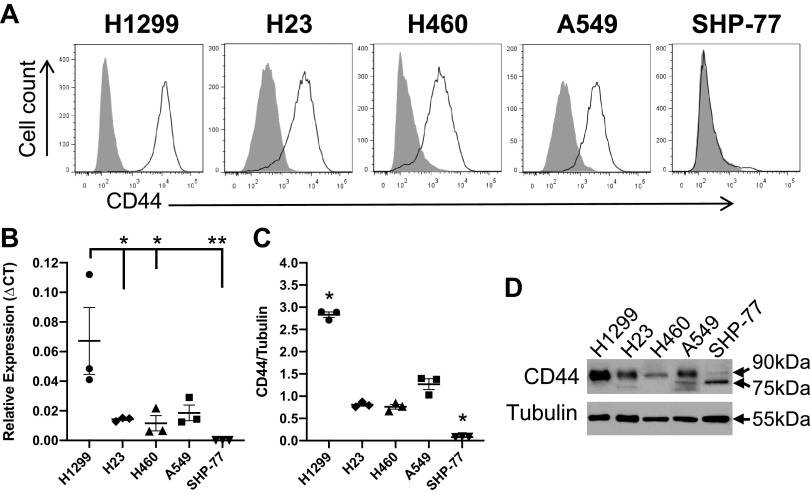
Lung cancer cells were probed for CD44. *A*: cancer cells were labeled with anti-CD44 (2C5) mAb (open curves) or isotype control (filled curves) and analyzed by flow cytometry. All cell lines, except for the SHP-77, positively expressed CD44. *B*: qRT-PCR was performed on H1299, H23, H460, A549, and SHP-77 mRNA using primers designed for the detection of CD44 standard. Data were analyzed using the Pfaffl method and presented as relative expression compared with the ribosomal protein L13A (RPL13A) housekeeping gene. CT is the threshold cycle. Data shown are means ± SE of *n* = 3 independent experiments with three technical replicates per experiment. Statistical analysis was performed by one-way ANOVA with Tukey’s HSD post hoc test. *P < 0.05 vs. H1299 (*P* = 0.035 for H23, *P* = 0.026 for H460). **P < 0.01 vs. H1299 (*P* = 0.008 for SHP-77). The results for all other comparisons were above the significance threshold (*P* < 0.05). *C*: cell lysate from 10^6^ cells was blotted with anti-CD44 (2C5) mAb. Tubulin loadings were used as controls for cell lysates. Data are representative of *n* = 3 independent experiments. Statistical analysis was performed by one-way ANOVA with Tukey’s HSD post hoc test comparison. *Significant differences compared with the other cell lines (*P* < 0.05). *D*: representative blot corresponding to *C*. HSD, honest significant difference.

Upon cholesterol depletion, surface protein shedding may occur (31), leading to a potential alteration of E-selectin ligand activities. Therefore, cell surface CD44 expression of the cells was examined after the treatments using flow cytometry. Cells were incubated with CD44 (2C5) mAb, which recognizes all CD44 isoforms. Flow cytometric analysis showed that all the NSCLC cell lines robustly expressed CD44, whereas the only SCLC cell line, SHP-77, failed to show any detectable CD44 expression (Fig. 5*A*). In addition, to quantitatively compare the expression of CD44 at the mRNA and protein level, qRT-PCR and Western blot analysis were performed. Both analyses showed that the H1299 cell line expressed higher levels of CD44 compared with the other lung cancer cell lines. In contrast, the SCLC line expressed a very low level of CD44 (Fig. 5, *B* and *C*), consistent with the results of surface protein expression by flow cytometry. In the Western blots, a band corresponding to the molecular weight of CD44s was observed around ∼90 kDa for H23, H1299, H460, and A549 cell lines. For the SHP-77 cell line, a thin band relative to the control with molecular weight of ∼75 kDa was detected (Fig. 5, *C* and *D*), corresponding to the soluble form of CD44 (45–49).

### Cholesterol Depletion Altered the CD44 Expression of NSCLC Cells

**Figure 6.**
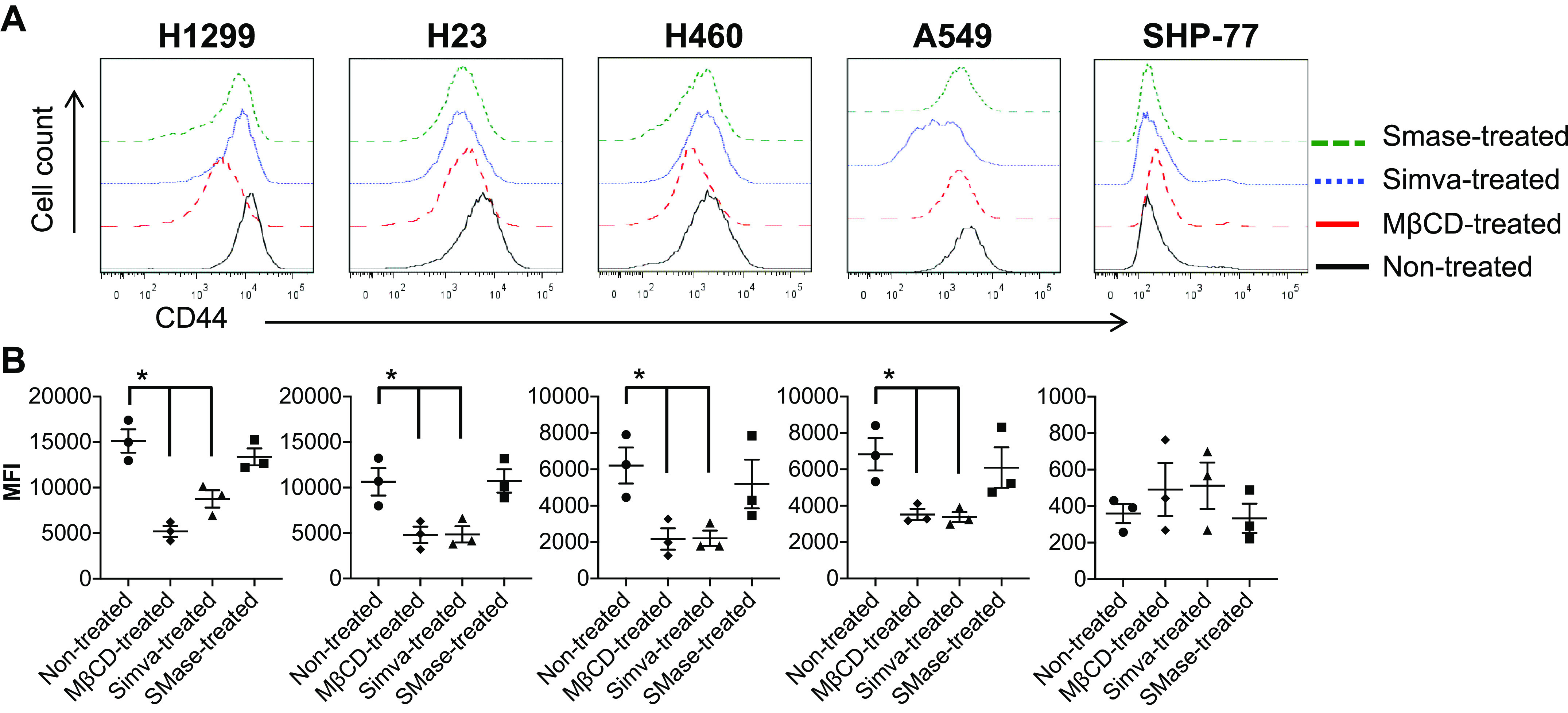
CD44 expression of lung cancer cells after mβCD, Simva, and SMase treatments. *A*: upon treatments with mβCD and Simva, CD44 was cleaved from the surface of almost all the NSCLC cells except for the SCLC cell line (with no CD44 expression; *A*). Histograms are representative of *n* = 3 independent experiments. *B*: mean fluorescence intensity (MFI) of CD44 expression showed a decrease for all NSCLC cell lines with mβCD and Simva treatments, whereas no change was observed for the SCLC cell line before and after treatments. Values are means ± SE for *n* = 3 independent experiments. Statistical analysis was performed by one-way ANOVA with post hoc Tukey’s HSD test comparison. *Statistical significance compared with nontreated control cells (*P* < 0.05). HSD, honest significant difference; MβCD, methyl-beta-cyclodextrin; NSCLC, non-small cell lung cancer; SCLC, small cell lung cancer.

Cells were treated with MβCD, Simva, or SMase to determine the effect of cholesterol depletion on CD44 surface expression of lung cancer cells by flow cytometry. As Fig. 6 shows, MβCD and Simva successfully lowered CD44 protein levels from the surface of all the NSCLC cell lines, but not for the SCLC cell line. Taken together with the results of the E-selectin adhesion assays, these findings imply that surface protein level changes induced by cholesterol depletion can impact the adhesive behavior of the cells.

### Cholesterol Content Regulates Plasma Membrane Fluidity

**Figure 7.**
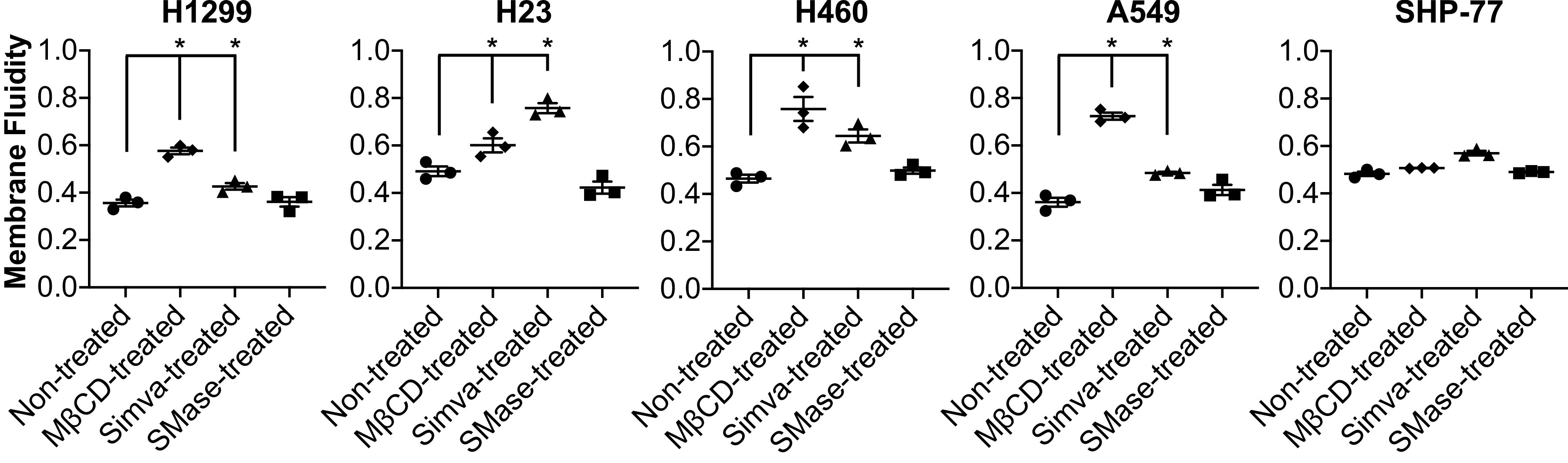
Membrane fluidity of nontreated and treated lung cancer cells estimated using the fluorescent probe PDA. Results are means ± SE of *n* = 3 independent experiments. Statistical analysis was performed by one-way ANOVA with post hoc Tukey’s HSD test. *Significant difference in comparison with nontreated cells (*P* < 0.05). HSD, honest significant difference; PDA, pyrenedecanoic acid.

The majority of cellular cholesterol is found in the plasma membrane (50, 51). To understand the observed differences in the dynamics of lung cancer cell attachment and rolling velocity, the plausible changes in membrane properties due to cholesterol depletion were investigated. We have previously shown that whole cell fluidity correlates with the cell attachment and rolling velocity (41); thus, the lung cancer cell membrane fluidity changes induced by MβCD, Simva, and SMase treatments were examined. As shown in Fig. 7, membrane fluidity of all the NSCLC cells increased upon cholesterol depletion through MβCD and Simva (but not SMase) treatments relative to the nontreated control cells. None of the treatments affected the lone SCLC cell line, SHP-77.

## DISCUSSION

Cholesterol has been accredited to be a major constituent in preserving the regulation of cellular processes as well as maintaining the structural integrity and membrane fluidity of the cells (31, 52–54). In the present study, we investigated the effect of the plasma membrane cholesterol alteration through MβCD, Simva, and SMase on cell adhesion and rolling of multiple NSCLC cell lines and an SCLC cell line on E-selectin coated flow chambers. More proliferative cancer cells have an increased cholesterol uptake in the plasma membrane compared with noncancerous cells (53, 55). Measuring the membrane cholesterol content of our panel of lung cancer cell lines, NSCLC cell lines showed significantly higher membrane cholesterol content compared with the SCLC cell line (Table 1). However, considering the surface area of the cells, it was noted that the SCLC cells, with at least a twofold smaller diameter, contain a comparable amount of cholesterols per surface area to the NSCLC cells (Fig. 2).

Accumulated cholesterol in lipid microdomains of proliferative cancer cells modulates cell adhesion and rolling on E-selectin (40). E-selectin has been reported to modulate the initial tethering and rolling of lung cancer cells on the endothelium (56). One of the E-selectin ligands extensively expressed on lung cancer cells, CD44 glycoprotein (11, 12), has been shown to be localized in the lipid microdomains of the cell membrane (17). Thus, the E-selectin ligand activity of the lung cancer cell lines was tested by perfusing the cells over E-selectin-coated surfaces under physiological flow conditions in the parallel-plate flow-chamber adhesion assay. The number of attached cells was significantly greater for the highly expressing CD44 protein NSCLC cell line, H1299, compared with the other cell lines (Figs. 4 and 5). Interestingly, for the SCLC cell line (SHP-77), which lacked CD44 protein expression, cell attachment was statistically higher compared with the NSCLC cell lines, excluding the H1299. In addition, all the NSCLC cell lines highly expressed sLe^X^ (Fig. 3), a common carbohydrate epitope for the three members of the selectin receptor family (E-, P-, and L-selectin) (57). Sialofucosylated glycoforms of CD44 have been shown to act as shear-resistant ligands for E-selectin in breast cancer cell adhesion to the endothelium (10), colon cancer cell lines (58, 59) as well as prostate cancer cell lines (60), but not yet on lung cancer cell lines to the best of our knowledge. Thus, work to identify the NSCLC and SCLC E-selectin ligands, including CD44 or other glycoconjugates, is currently ongoing in our laboratories.

Upon cholesterol depletion and inhibition through MβCD and Simva treatments, membrane cholesterol content per surface area of all the studied lung cancer cell lines significantly decreased (two to threefold), whereas SMase did not show much potency for cholesterol depletion (Fig. 2). SMase has shown to hydrolyze sphingomyelin from sphingomyelin-cholesterol microdomains within the plasma membrane and result in a rapid translocation of the cholesterol to endoplasmic reticulum (ER) (61). However, Porn et al. (62) showed that the overall membrane cholesterol of SMase-treated cells did not change, suggesting a transient cholesterol relocation and immediate retention of the majority of cholesterol to the cell membrane. Thus, sphingomyelin breakdown through SMase may alter the cholesterol distribution on the cell membrane, but it may not change the cholesterol content (63–65).

The results of flow chamber cell adhesion assay showed that the number of attached NSCLC cells significantly decreased (two to sevenfold) through cholesterol depletion and inhibition by MβCD and Simva treatments, whereas the number of attached cells remained unaffected after SMase treatment (Fig. 4*A*). Interestingly, the number of attached SCLC cells was not altered after any of the treatments (Fig. 4*A*). Moreover, the rolling velocities of the H1299 and H23 cells increased after MβCD-cholesterol-depletion, whereas the rolling velocity of the SHP-77 (SCLC) cells stayed unaffected (Fig. 4*B*). Moreover, CD44 expression of almost all the NSCLC cell lines, but not the CD44 negative SCLC cell line, decreased at least twofold at the molecular surface level after treating the cells with MβCD and Simva (Fig. 6). The enhanced CD44 shedding through cholesterol depletion has been reported to be mediated by a disintegrin and metalloproteinase 10 (ADAM10) as a result of disintegration of cholesterol-rich microdomains, and thus CD44 disordered localization within the plasma membrane (31).

Membrane fluidity or cell deformability is known to regulate the rolling and adhesion of cells to the stimulated vascular endothelium (40, 66, 67). Gimpl et al. (52) have suggested that since cholesterol acts as the main lipid rigidifier in the membrane, cholesterol depletion results in greater membrane fluidity. To further investigate the effect of cholesterol depletion on membrane fluidity and thus cell adhesion, we measured the alternations in membrane fluidity of the cells after treatments. Membrane fluidity of NSCLC cells increased around 1.5–2-fold upon cholesterol depletion with MβCD and Simva, with a 1.5–2.5-fold reduction in cholesterol content per surface area. However, the membrane fluidity of NSCLC cells did not change by SMase treatment (Fig. 7), as SMase also did not reduce the cholesterol content. However, for the SHP-77 cells without CD44 expression, despite the observed cholesterol reduction through MβCD and Simva treatments (Fig. 2), the membrane fluidity of this SCLC cell line remained unaffected (Fig. 7). Moreover, we have previously shown a correlation between CD44 and membrane fluidity in breast cancer cells (41). In the breast cancer cell lines we studied, a negative shift in CD44 expression was observed upon long-term treatment with cytochalasin D (41). Cytochalasin D, an inhibitor of actin polymerization, can lead to a rapid decrease in the viscosity (increase in the fluidity) of cell membranes (68). Therefore, the enhanced membrane fluidity of the NSCLC cells after treatments with MβCD and Simva could be the result of CD44 shedding induced by membrane cholesterol depletion (31, 40). In addition, we have shown that breast cancer stem cells with enhanced fluidity (lower viscosity and cortical tension) have a more stable shear-independent rolling behavior under physiological flow conditions compared with non-stem breast cancer cells (41). More-stable cell rolling on endothelial cells may facilitate the firm adhesion bonds and thus raise the cells’ metastatic potential (40, 69).

The adhesion of the SHP-77 cells to E-selectin was not mediated by cholesterol content nor correlated to CD44, thus the number of attached SCLC cells and their membrane fluidity remained constant after the cholesterol depletion treatments. Therefore, for the SCLC cells, with their relatively high cholesterol per volume and lack of surface CD44 expression, the molecular and biophysical mechanisms of cell adhesion and migration are likely to be different from those of the NSCLC cells. We hypothesize that the diameter of the tumor cells might be a critical factor in cell adhesion and rolling under physiological flow conditions (70). Because of their dimensions, not only small cancer cells might be able to easily intravasate into the bloodstream to begin the metastatic invasion, but they might also have the advantage of not being pushed away by the shear flow compared with larger cells that experience greater torque (71, 72). This can also explain the significantly slow rolling velocity of the SCLC cells on E-selectin compared with the NSCLC cell lines (Fig. 4*B*). Therefore, small cells with relatively low rolling velocities might have a higher chance to initiate firm adhesion to endothelial cells, which eventually leads to extravasation out of the blood vessel. However, the proposed benefit of better attachment for small cells might also be a function of shear stress, thus more investigation is needed to understand the significance of NSCLC and SCLC cell sizes in their adhesion capability under different flow rates.

In summary, we have demonstrated that altering CD44 distribution and membrane fluidity through cholesterol depletion modulates the adhesion and rolling of NSCLC cells on E-selectin under physiological conditions. Based on these findings, we might envision the potential therapeutic approach of targeting cholesterol depletion in NSCLC cells to inhibit cancer metastasis. However, the enhanced cell membrane fluidity in circulating NSCLC cells may cause a more stable shear-independent rolling on endothelium, resulting in firm adhesions and thus promoting metastasis. Our work highlights the importance of further study in biophysical regulation by cholesterol levels as another possible mechanism in addition to the known biochemical pathways in the pathophysiology of the metastatic process.

## DATA AVAILABILITY

Data will be made available upon reasonable request.

## GRANTS

This work was supported by the Russ College of Engineering and Technology at Ohio University, grants from the National Institutes of Health R01-DK054254, R01-DK083850, and R01-HL112248 (to S.M.N.), and a grant from the SLU President’s Research Fund PROJ-000248 (to A.M.).

## DISCLOSURES

No conflicts of interest, financial or otherwise, are declared by the authors. 

## AUTHOR CONTRIBUTIONS

A.M. and M.M.B. conceived and designed research; A.M., C.A.S., and H.T.M. performed experiments; A.M., C.A.S., and H.T.M. analyzed data; A.M., C.A.S., H.T.M., A.M.F., S.M.N., and M.M.B. interpreted results of experiments; A.M. prepared figures; A.M. drafted manuscript; A.M., C.A.S., H.T.M., A.M.F., S.M.N., and M.M.B. edited and revised manuscript; A.M., C.A.S., H.T.M., A.M.F., S.M.N., and M.M.B. approved final version of manuscript. 
